# Prospective Observational Evaluation of Fixed Combination Calcipotriol/Betamethasone Aerosol Foam (Enstilar®) in the Management of Psoriasis with Scalp Involvement in Everyday Clinical Practice (the CAPITIS Study)

**DOI:** 10.1159/000527496

**Published:** 2023-01-30

**Authors:** Petra Staubach, Elisabeth Wurzer, Hans Joachim Hutt, Ralph von Kiedrowski

**Affiliations:** ^a^Department of Dermatology and Allergy, University Medical Center, Mainz, Germany; ^b^LEO Pharma GmbH, Neu-Isenburg, Germany; ^c^Dermatologische Spezialpraxis für chronisch-entzündliche System-Dermatosen, Dermato-Onkologie und Allergologie, Selters (Westerwald), Germany

**Keywords:** Aerosol foam, Betamethasone, Calcipotriol, Scalp psoriasis

## Abstract

**Background and aim:**

Clinical trials have demonstrated the efficacy of fixed-dose combination calcipotriol/betamethasone (Cal/BD) aerosol foam for the treatment of patients with scalp psoriasis. However, data on the real-world effectiveness of Cal/BD aerosol foam in this subgroup of patients are lacking. Therefore, this study investigated the effectiveness and tolerability of 4 weeks' treatment with Cal/BD aerosol foam in patients with scalp psoriasis in everyday clinical practice.

**Methods:**

This prospective, non-interventional multicenter study involved 217 adults with scalp psoriasis who were treated with Cal/BD aerosol foam for 4 weeks. Primary endpoints included the proportion of patients with <10% of the scalp area affected (Scalp-BSA) plus a Scalp-PGA of “mild” after 4 weeks, as well as the proportion of patients with an absolute PSSI ≤2 points after 4 weeks. Secondary endpoints included patient reported changes in erythema, itching, flaking, and thickness at baseline, 3 days, 1 week, 2 weeks, and 4 weeks.

**Results:**

After 4 weeks, 53.4% of patients treated with Cal/BD aerosol foam had achieved a Scalp-BSA of <10% and a mild Scalp-PGA. Furthermore, 47.6% of patients achieved a PSSI ≤2. Improvements in pruritus and other symptoms (induration, erythema, and scaling) were seen already within 3 days. The proportion of patients who reported that scalp psoriasis had no influence on their quality of life (Dermatology Quality of Life Index 0/1 points) increased from 3.2% at baseline to 47.9% at study end. Patient satisfaction with treatment was high (Treatment Satisfaction Questionnaire-9 scores of 74.5 ± 27.1 for effectiveness, 72.0 ± 25.2 for ease of use, and 77.8 ± 24.2 for general satisfaction). Overall, 97.4% of HCPs assessed the tolerability of Cal/BD aerosol foam as good/very good with no new safety concerns.

**Conclusion:**

This study demonstrated the effectiveness, rapid onset of action, good tolerability, and good safety profile of the Cal/BD aerosol foam in patients with scalp psoriasis treated in a real-world setting.

## Introduction

Psoriasis is a chronic inflammatory dermatological disease characterized by sharply demarcated erythematous plaques with a thick silvery-white scale [1]. The scalp is often the first skin area to be affected by psoriatic lesions. It remains the most common site of disease involvement throughout the patient's life but is considered a difficult-to-treat area. It poses some challenges to topical formulations due to the hairiness of the affected area [2]. The severity of scalp psoriasis can range from mild (slight fine scaling, with symptoms akin to dandruff) to extensive/severe (where large areas of the scalp are covered in thick crusted plaques), and it often coexists with psoriasis on other parts of the body [2, 3]. Scalp psoriasis is associated with a triad of debilitating symptoms which include intense pruritus, soreness, and scaling/shedding and, in addition to these, temporary hair loss and scarring alopecia have also been reported. However, the latter appear to be relatively less common [1, 2, 3, 4].

From the patient's perspective, the stigmatization resulting from scalp psoriasis has been reported to have a marked deleterious effect on psychosocial functioning, with about one-third of affected patients indicating that it caused them some level of distress [1]. As a consequence, the physical and psychosocial impact of scalp psoriasis is significant, and published reports on this topic highlight the negative effects it has on the quality of life (QoL) of the individual [1, 4, 5]. It may be further exacerbated if the lesions extend beyond the scalp onto the neck, forehead, and ears which is often the case and typical for psoriasis vulgaris. Major involvement of visible areas as well as the scalp have been defined as a possible criterion to “upgrade” the severity of psoriasis from mild to moderate to severe disease [6].

It has been estimated that as many as 80% of patients with psoriasis have some degree of scalp involvement [7], and the prevalence of scalp psoriasis has been reported to range between 1 and 2%, both in North America [8] and Europe [1, 9]. Overall, approximately 80% of all patients with psoriasis can be adequately treated using topical therapy [10, 11]. The clinical management of patients with scalp psoriasis is largely dictated by the severity of the disease and the challenges posed by the presence of hair; topical therapies are the mainstay of treatment for patients with mild to moderate disease [4]. Fixed-dose combination therapy with a vitamin D analogue and a corticosteroid, such as calcipotriol/betamethasone (Cal/BD), is the gold standard in topical psoriasis therapy [12]. Combining both active ingredients has synergistic effects against the pathophysiological mechanisms underlying psoriasis while attenuating the side effects. Steroids are immunosuppressive and inhibit the proinflammatory environment and T-cell activation, while vitamin D analogues reduce hyperproliferation and abnormal differentiation of keratinocytes, as well as having immunomodulatory effects [13]. Clinical trials support the superior efficacy and good tolerability/safety of fixed-dose combination Cal/BD aerosol foam compared with the individual monotherapies for treatment of flares, including in patients with scalp psoriasis [13, 14]. They have also shown the safety as well as the efficacy of Cal/BD aerosol foam in long-term proactive management [15].

Clinical trials involve a selected patient population managed in a controlled environment, and it is important to also obtain data from patients treated in everyday clinical practice [16]. There is real-world evidence supporting the effectiveness of fixed-dose combination Cal/BD aerosol foam in patients with plaque psoriasis [16, 17], but there is a lack of real-world data specifically in patients with scalp psoriasis. The current study was the first real-world study to evaluate this aspect of care. The aim of this non-interventional study was to investigate the effectiveness and tolerability of 4 weeks' therapy with Cal/BD aerosol foam (Enstilar®) for treating patients with signs and symptoms of psoriasis vulgaris with scalp involvement in everyday clinical practice.

## Methods

### Study Design

This was a prospective, non-interventional, observational multicenter study with two visits (baseline and ∼4 weeks) which planned to include approximately 500 adult patients with psoriasis vulgaris with scalp involvement from between December 15, 2019, and June 30, 2020. The study was conducted in compliance with the German Medicinal Products Act (AMG, § 4 (23), sentence 3), was approved by the Ethics Committee of Landesärztekammer Rheinland-Pfalz, and the positive decision was notified to the respective German regulatory bodies.

### Change to Protocol

The execution of this non-interventional study coincided with the escalation of the SARS-CoV-2 (Covid-19) pandemic which had a marked impact on patient recruitment. The initial goal was to document 500 adult patients with scalp psoriasis between December 2019 and June 2020. When it was clear that this was not achievable, the goal of the study was modified to include >200 patients, and the recruitment phase was extended by an additional 2 months.

### Treatment

Calcipotriol 50 μg/g/betamethasone dipropionate 0.5 mg/g aerosol foam (Cal/BD aerosol foam; Enstilar®) was applied once daily to the affected scalp for 4 weeks in accordance with the recommendations provided in the approved SmPC.

### Patients

Key inclusion criteria: male or female adults (>18 years-old) with confirmed psoriasis vulgaris and scalp involvement; ≥10% of the scalp area and a plaque severity of at least mild (≥2 points on a Scalp Physician Global Assessment [Scalp-PGA] using a 5-point scale ranging from 0 to 4) or at least 2 of 3 symptoms (erythema, induration, scaling) within the Psoriasis Scalp Severity Index (PSSI) rated at least mild (≥1-point using 5-point scales each ranging from 0 to 4). Patients for whom treatment with Cal/BD aerosol foam was foreseen, and where the decision to start treatment with Cal/BD aerosol foam was made independently and before inclusion into this non-interventional study, could participate. All patients voluntarily agreed to participate and had read and signed the informed consent form.

Key exclusion criteria were contraindications as per the SmPC or known allergies to the Cal/BD aerosol foam or one of its ingredients; treatment with systemic psoriasis therapy or UV therapy within the 2 weeks prior to enrolment, severe (>30% of body surface area [BSA]), or other types of psoriasis (e.g., erythrodermic psoriasis or pustular psoriasis); or enrolment in another interventional study. Patients previously treated with Cal/BD aerosol foam within 3 months before the start of the study were also excluded.

### Procedures

#### Assessment by Investigators

At the baseline visit, investigators documented patient characteristics (demographics, socioeconomic characteristics, and general medical history), psoriasis history, current status of scalp psoriasis, and scalp psoriasis treatment (including reasons for choosing Cal/BD aerosol foam and planned frequency). Scalp psoriasis was assessed using Scalp-BSA (percentage of affected scalp area), Scalp-PGA (categories were defined as “mild/moderate/severe” [5], where the category “mild” was the lowest possible category to rank and thus also included “clear” lesions), and PSSI (affected scalp area, average degree of scalp erythema, induration, and desquamation severity; score ranging from 0 to 72 [18]). After approximately 4 weeks' treatment, the following were recorded at a second clinic visit: current status of scalp psoriasis (Scalp-BSA, Scalp-PGA, and PSSI); reported or observed adverse drug reactions; final assessment of effectiveness, safety, and satisfaction with Cal/BD aerosol foam.

#### Patient Questionnaires

At baseline, during and at the end of the study, patients were asked to complete questionnaires regarding their psoriasis, its treatment, QoL, satisfaction with previous and current treatments, and details of symptoms and any changes in disease severity. The following questionnaires were completed. (1) At baseline: Dermatology Quality of Life Index (DLQI); assessment of the severity of scalp psoriasis with Scalp-PaGA (Scalp patients' global assessment using a 6-point scale of 0 = clear to 5 = very severe); scalp symptoms such as erythema, flaking, and thickness; lesion visibility (using a 5-point scale of 0 = not at all to 4 = very); itch (using a numeric rating scale [NRS] ranging from 0 = no itch to 10 = maximum itch); as well as psoriasis spread (categories: 0 = 0%, 1 = <10%, 2 = 10–29%, 3 = 30–59%, 4 = 60–89%, 5 = 90–100%). (2) Follow-up questionnaires: date treatment started; details on any other topical treatment for scalp psoriasis; perception of the treatment (soothing, cooling, antipruritic) on day 3 (each rated using a 5-point scale ranging from “very strong” to “not at all”); assessment of the severity of scalp psoriasis (Scalp-PaGA, scalp symptoms, itch, as well as psoriasis spread as described for baseline) on day 3 and after 1 and 2 weeks' treatment. (3) After approximately 4 weeks of treatment: severity of scalp psoriasis (Scalp-PaGA, scalp symptoms, itch, and psoriasis spread as described for baseline); treatment strategies for Cal/BD aerosol foam application (e.g., frequency, number of doses); DLQI; and a Treatment Satisfaction Questionnaire (TSQM-9).

### Outcomes

#### Primary endpoints

The proportion of patients with <10% of the scalp area affected (Scalp-BSA) plus a Scalp-PGA of “mild” after 4 weeks, as well as the proportion of patients with an absolute PSSI ≤2 points after 4 weeks.

#### Secondary endpoints

The mean improvement in affected scalp area (Scalp-BSA) and psoriasis severity (Scalp-PGA) after 4 weeks, the proportion of patients who assessed the severity of their scalp psoriasis as “clear/almost clear” (Scalp-PaGA = 0/1, 6-point scale) after 4 weeks, the proportion of patients with no or mild itching on the scalp, which was rated 0–2 points (using a NRS of 0–10) after 4 weeks, and mean improvement in patient reported psoriasis symptoms including, itching (NRS, 0–10), erythema, flaking, thickness, lesion visibility (5-point scale, 0–4), and affected scalp area (6-point scale, 0–5) overtime (at baseline, and after ∼3 days, and ∼1, ∼2, and ∼4 weeks).

### Statistical Analyses

In this non-interventional study, statistical analyses were descriptive and explorative, and no starting statistical hypothesis was included in the protocol. The study populations included in the analyses were: (a) the intention-to-treat (ITT) population which included all patients who had provided written informed consent for the use of their data in this study; (b) the safety population which was a subgroup of the ITT population and only included patients who had applied the Cal/BD aerosol foam at least once; and (c) the effectiveness population which was a subgroup of the safety population and included all patients for whom at least one effectiveness variable had been documented.

For quantitative variables, mean, standard deviation, quartiles, and minimum/maximum values were calculated. For nominal and ordinal variables, absolute and relative frequencies were calculated. Statistical significance will be presented for important parameters but shall be interpreted in a descriptive-exploratory way only.

## Results

A total of 217 patients from 96 German centres were enrolled in this non-interventional study (ITT population), and 201 patients were included in the effectiveness and safety populations.

### Patient Demographics/Characteristics

Male or female adult patients with a mean ± SD age of 49.6 ± 17.2 years were enrolled into the study, and the main sociodemographic characteristics, psoriasis history, and comorbidities are shown in Table 1. Lack of effectiveness of the previous therapy was the major reason for choosing Cal/BD aerosol foam (135 patients; 64.6%), and 94.9% of the patients were prescribed to apply the treatment at night, 3.7% in the morning, and 1.4% three times per week. All patients confirmed that they had been informed about how to use Cal/BD aerosol foam and had received the Enstilar® package leaflet. During the study, 69.7% of patients who reported on their application regimen (*n* = 188) applied Cal/BD aerosol foam once daily, while 6.4% applied it 4–6 days per week, 19.1% applied it 1–3 days per week, and 6.9% applied it as needed.

### Physicians Assessments

#### Scalp Psoriasis Status

**Fig. 1:**
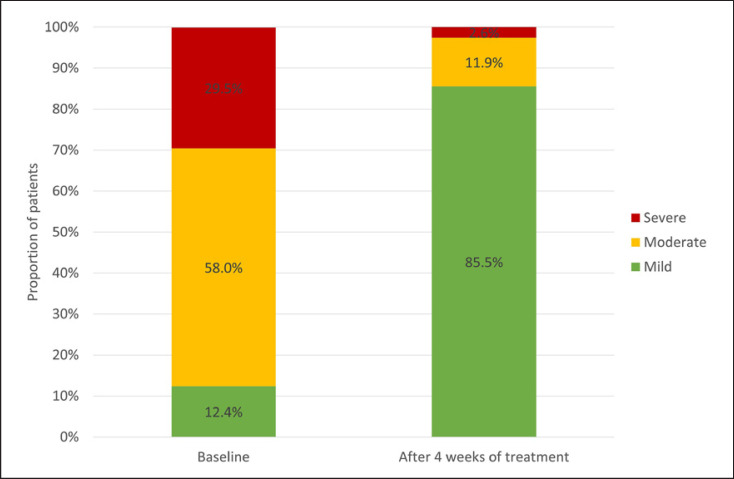
Scalp psoriasis status measured using the Scalp Physician Global Assessment (Scalp-PGA) at baseline and after 4 weeks' treatment with Cal/BD aerosol foam (*n* = 193); Scalp-PGA was assessed with the three categories “mild/moderate/severe” [5], with the category “mild” being the lowest possible category to rank, thus also including “clear” lesions.

At the completion of this observational study, 53.4% of patients (*n* = 103) treated with Cal/BD aerosol foam had achieved a Scalp-BSA of <10% of the affected scalp area and a mild Scalp-PGA (primary endpoint). The mean ± SD Scalp-BSA decreased during treatment from 32.5 ± 19.0% pretreatment to 10.9 ± 12.5% after 4 weeks (*p* < 0.001). The mean change in scalp psoriasis status after 4 weeks' treatment with Cal/BD aerosol foam, as measured using the Scalp-PGA, is shown in Figure 1. The proportion of patients with a mild status increased from 12.4% (24 patients) to 85.5% (165 patients), while the proportion of patients with moderate or severe rating decreased from 58.0% (112 patients) and 29.5% (57 patients) to 11.9% (23 patients) and 2.6% (5 patients), respectively. Furthermore, 47.6% of patients (*n* = 91) achieved a PSSI ≤2 points after 4 weeks' treatment with Cal/BD aerosol foam (primary endpoint). The mean PSSI decreased from 19.74 ± 11.68 at baseline to 4.75 ± 5.72 after 4 weeks (*p* < 0.001).

### Patient Assessments

#### Scalp Psoriasis Status: PaGA

**Fig. 2:**
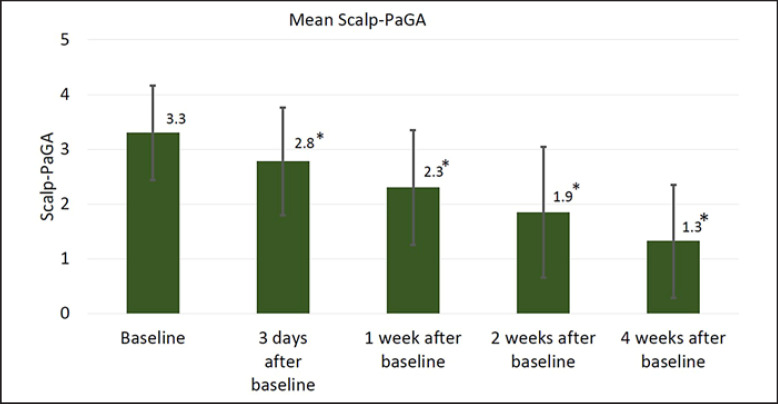
Severity of scalp psoriasis during the course of the 4-week study measured using the scalp psoriasis patient global assessment (Scalp-PaGA, 0 = clear, 5 = very severe; *n* = 193; LOCF; mean ± SD); *p < 0.001 versus baseline.

**Fig. 3:**
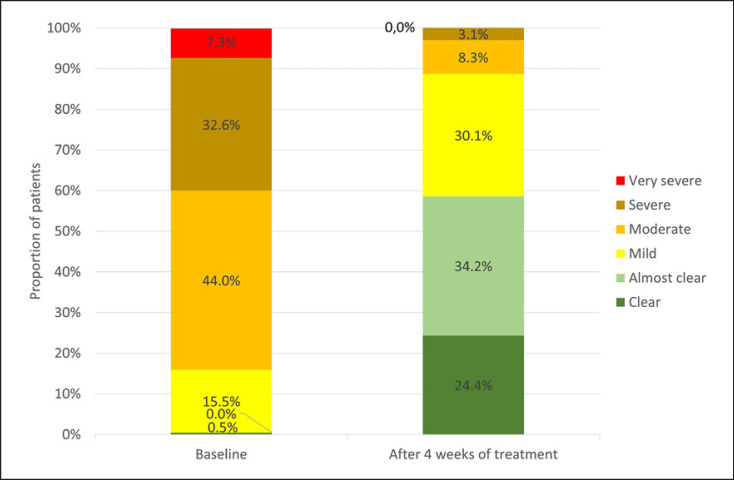
Severity of scalp psoriasis during the course of the 4-week study measured using the scalp psoriasis patient global assessment (Scalp-PaGA) (*n* = 193).

The Scalp-PaGA demonstrated that patients' perceived severity of the condition improved continuously during 4 weeks' treatment with Cal/BD aerosol foam. The mean ± SD rating at baseline was 3.3 ± 0.9 points, and this had markedly decreased to 1.3 ± 1.0 points (last observation carried forward) by the end of the study (Fig. 2) (*p* < 0.001). At the start of the study, 83.9% patients rated their scalp psoriasis as moderate/severe/very severe, and this was reduced to 11.4% after 4 weeks' treatment with Cal/BD aerosol foam (Fig. 3). Conversely, at the start of the study, the scalp psoriasis was rated as clear/almost clear to mild by 0.5% and 15.5% of patients, respectively, and these numbers had increased to 58.6% for clear/almost clear and 30.0% for mild after 4 weeks' treatment.

**Fig. 4:**
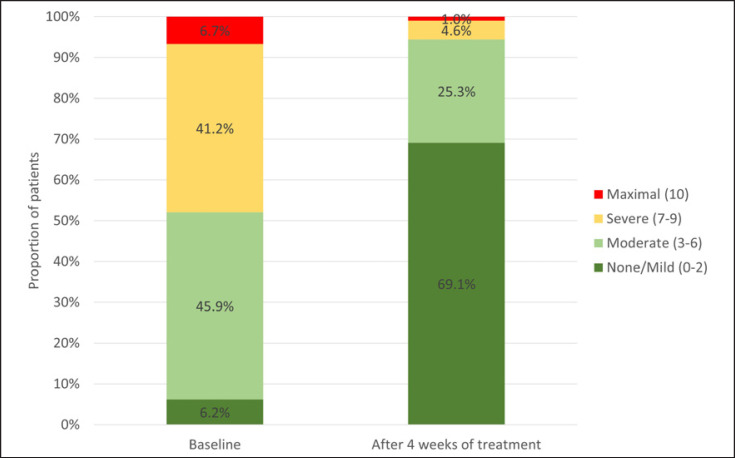
Patient reported perception of itch on the affected scalp rated using a NRS ranging from 0 = no itch to 10 = maximum itch at baseline and after 4 weeks of treatment with Cal/BD aerosol foam (*n* = 194).

Patients rated the severity of their scalp psoriasis according to 6 main criteria: flaking, erythema, thickening, visibility, psoriasis spread and itch, and the changes during the trial (baseline, 3 days, 1 week, 2 weeks, and end of study) are shown in Table 2. For all parameters, the severity of symptoms decreased significantly as early as day 3 and continued to improve throughout the study. Notably, the score for itch decreased from 6.1 ± 2.2 at baseline to 4.9 ± 2.4 after 3 days' treatment (*p* < 0.001) and 2.0 ± 2.2 after 4 weeks (*p* < 0.001). Furthermore, 69.1% of patients reported no or only mild itching on the scalp (0–2 points using a NRS [0–10]) after 4 weeks of treatment with Cal/BD foam compared to 6.2% at baseline (Fig. 4).

### Quality of Life

During the current study, the QoL of patients improved significantly during treatment with Cal/BD aerosol foam. The mean ± SD DLQI score before the start of treatment was 9.6 ± 5.5, and this was reduced to 2.8 ± 3.5 at the end of the study (*p* < 0.001). This shows an overall improvement in the QoL of patients with psoriasis who were treated with Cal/BD aerosol foam for 4 weeks.

**Fig. 5:**
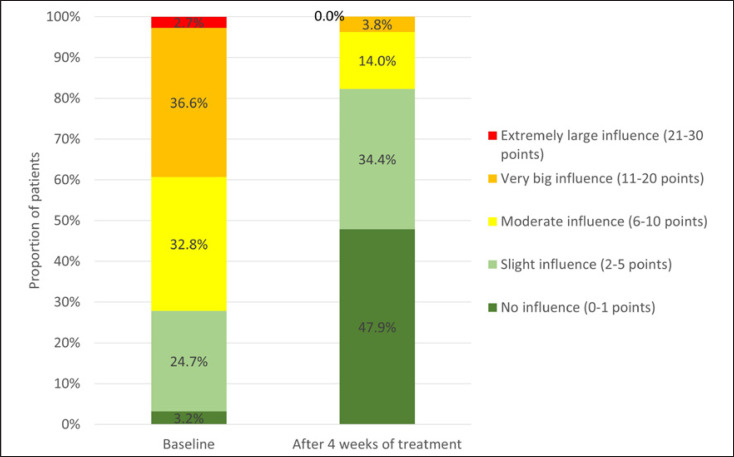
Influence of scalp psoriasis on the QoL of patients measured using Dermatology Quality of Life Index (DLQI) at the start of therapy and end of study following 4 weeks' treatment with Cal/BD aerosol foam (*n* = 186).

For most patients questioned, QoL improved markedly. At the beginning of the study, the majority of respondents (36.6%) indicated that scalp psoriasis had a very large influence on the QoL, whereas a small, moderate, or extremely large influence on their QoL was reported by 24.7%, 32.8%, and 2.7%, respectively. At the end of the study, QoL had markedly improved, with only 3.8% of patients reporting that scalp psoriasis still had a very large influence on their QoL (baseline: 36.6%). The number of patients in whom psoriasis had an extremely large impact on QoL decreased to 0 versus 5 patients at the beginning of the study. Importantly, the proportion of patients who reported that their scalp psoriasis had no influence on their QoL (DLQI 0/1 points) increased from 3.2% at baseline to 47.9% at study end (Fig. 5).

### Treatment Satisfaction

**Fig. 6:**
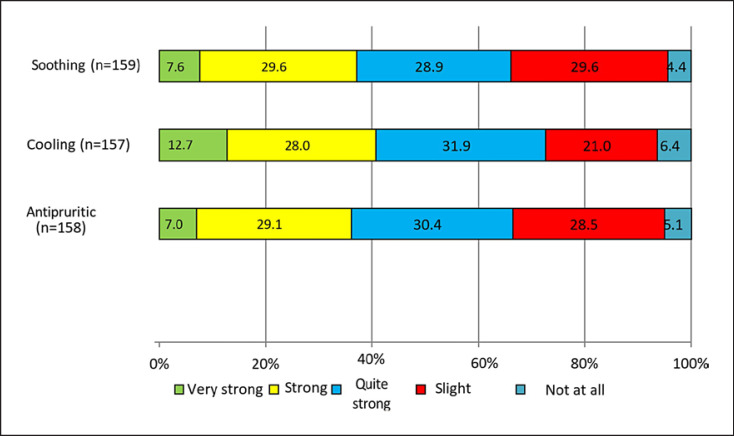
Patient reported perception of Cal/BD aerosol foam feeling on the skin at day 3 (each category rated using a 5-point scale ranging from “very strong” to “not at all”).

Patient perception of the effects of Cal/BD aerosol foam on the skin was assessed in a questionnaire completed by patients on day 3 of treatment. Properties of the foam aligned to the treatment of psoriasis were investigated, and >60% of patients reported quite strong, strong, or very strong soothing, cooling, and antipruritic properties (Fig. 6).

After the 4-week study, physicians rated the effectiveness of Cal/BD on the scalp as very good or good for 92.7% of patients (very good/good/moderate/poor corresponding to 60.1%/32.6%/6.7%/0.5%, respectively) and the ease of use of Cal/BD on the scalp as very good or good for 79.3% of patients (very good/good/moderate/poor corresponding to 41.5%/37.8%/13.5%/7.3%, respectively). Using TSQM-9, overall patient satisfaction with Cal/BD aerosol foam therapy with respect to effectiveness (74.5 ± 27.1), ease of use (72.0 ± 25.2), and general satisfaction with the aerosol foam (77.8 ± 24.2) were all considered to be relatively high.

### Consumption

By the end of the study, most patients (82.2%) had opened 1 can of Cal/BD aerosol foam, while 15.1% had started to use a second can.

### Safety and Tolerability

For 1 patient (0.5%), two adverse events were reported. The adverse events “cardiac disorder” and “dizziness” were of mild intensity and not deemed to be related to treatment. A total of 69 patients (34.4%) reported other experiences such as “inappropriate timing of product administration” (*n* = 68, 98.6%), “therapeutic product effect incomplete” (*n* = 2, 2.9%), and “drug ineffective” (*n* = 1, 1.5%). The intent to continue therapy twice weekly or on demand after the end of the study period was recorded as “inappropriate timing of product administration,” as the SmPC did not yet include proactive management at the time the study was conducted. After 4 weeks, the tolerability of Cal/BD aerosol foam was assessed and rated as good to very good by 97.4% of physicians (*n* = 193) who completed a global assessment of tolerability questionnaire (very good/good/moderate/poor corresponding to 69.4%/28.0%/2.1%/0.5%, respectively).

## Discussion

This investigation represents the first non-interventional study to investigate the effectiveness, tolerability, and safety of Cal/BD aerosol foam in patients with scalp psoriasis in a real-world setting. In the current non-interventional study, more than 50% of patients achieved the primary endpoint, comprising a Scalp-BSA of <10% of the affected scalp area and a mild Scalp-PGA. The mean Scalp-BSA was also substantially decreased after 4 weeks' treatment. Another important finding in the current study was the increase in the proportion of patients whose disease was assessed as mild on the Scalp-PGA, which reached 85.5% after 4 weeks' treatment. Furthermore, almost half of the group achieved a PSSI ≤2 points after 4 weeks' treatment with Cal/BD aerosol foam (also a primary endpoint). The effectiveness reported by the physicians is in line with the patients' self-assessment, which shows an overall improvement in the severity of scalp psoriasis symptoms during the 4 weeks' treatment period, as indicated by the Scalp-PaGA results. The results highlight the good overall effectiveness of 4 weeks' treatment of scalp psoriasis in everyday clinical practice and are consistent with those from a randomized controlled clinical trial of Cal/BD aerosol foam in 302 patients with psoriasis [14]. In the latter randomized controlled clinical trial, application of Cal/BD aerosol foam resulted in treatment success in 26% of patients with scalp psoriasis as early as week 1 and 53% of patients with scalp psoriasis after 4 weeks, which was significantly higher than for Cal aerosol foam monotherapy (53.0% vs. 35.6; *p* = 0.021) [14]. However, it should be noted that the results of our study and those of Lebwohl and colleagues are not directly comparable since different scoring tools were used.

Symptoms typically observed in patients with scalp psoriasis such as flaking, erythema, plaque thickness, lesion visibility, psoriasis spread, and even itch were all markedly improved during the 4-week study, and clinical improvement was noted within 3 days after starting treatment. A fast onset of action was also observed in the phase 2 clinical trial involving patients with scalp psoriasis, with a higher effectiveness of Cal/BD aerosol foam versus the monotherapies seen as early as week 1 [14]. The rapid improvement in symptoms during treatment with Cal/BD aerosol foam, as well as the effective reduction in itch for the majority of Cal/BD aerosol foam patients in this study, are significant attributes which are of great benefit to patients given that chronic pruritus is often a particularly distressing problem for patients, causing sleep loss and reduced QoL [19].

The value of treatment with Cal/BD aerosol foam was also reflected in the patients' perception of their QoL, with a reduction in DLQI scores during the study. This corresponds to an overall improvement in QoL for patients with scalp psoriasis who were treated with Cal/BD aerosol foam for 4 weeks. For most patients, QoL improved markedly, with almost 50% of patients achieving a DLQI score of 0/1 at study end, which indicates that there is no impact of the dermatologic disease on the QoL. This is an important aspect in patient care, given the negative effect that psoriasis usually has on QoL and psychosocial well-being [20].

During the study, most patients applied Cal/BD once daily, and after 4 weeks, the majority had used 1–2 cans of Cal/BD, suggesting reasonable adherence to treatment. In general, poor adherence of patients to topical therapies is due to a combination of factors such as lack of effectiveness, time-consuming or burdensome to apply, poor cosmetic properties, or side effects [21, 22].

Besides the effectiveness of Cal/BD aerosol foam in reducing Scalp-BSA, PSSI, and increasing the numbers of patients with mild Scalp-PGA, the aerosol foam was well-tolerated and only two adverse events were reported, and these were not considered to be related to treatment. Insufficient effectiveness was documented in only 1 patient. Very good or good “easy to use on the scalp” was recorded by physicians for 79.3% of patients, and the patients themselves also rated the “ease of use” relatively highly (72.0 points in TSQM-9). Accordingly, the foam formulation of Cal/BD administered via an aerosol may contribute to improved adherence due to its good effectiveness and highly rated ease of application. At the end of the study period, 146 patients (74.1%) indicated that they wanted to continue treatment with Cal/BD aerosol foam, with most (53.4%) planning to use it once daily, 23.3% planning to use it twice per week, and 23.3% planning to use it as needed.

This non-interventional, observational study was designed to gather real-world data on the application of Cal/BD aerosol foam to the scalp of patients with scalp psoriasis and to describe the profile of a non-selected population of patients treated in everyday clinical practice, including those with comorbidities, receiving concomitant therapies, or with a history of previous topical treatment options. Overall, the study highlighted the clinical value of Cal/BD aerosol foam treatment in terms of its high effectiveness, rapid onset of action (improved scalp symptoms within 3 days), good tolerability (rated as good to very good by 97.4% of physicians), and good safety profile in patients with scalp psoriasis.

## Key Message

Cal/BD aerosol foam was rapidly effective and well-tolerated in patients with scalp psoriasis.

## Statement of Ethics

The study was conducted in compliance with the German Medicinal Products Act (AMG, § 4 (23), sentence 3), was approved by the Ethics Committee of Landesärztekammer Rheinland-Pfalz, and the positive decision was notified to the respective German regulatory bodies. Following detailed instructions of the aims of this non-interventional study, all patients voluntarily agreed to their participation and provided written informed consent for the use of their data from the study.

## Conflict of Interest Statement

Petra Staubach was an advisor/consultant for AbbVie, Allergika, Almirall Hermal, Amgen, Beiersdorf, BioCryst, Biogen Idec, BMS, Boehringer Ingelheim, Celgene, CSL Behring, Eli-Lilly, Galderma, Hexal, Janssen, Klinge, LEO Pharma, LETI Pharma, L´Oreal, Novartis, Octapharma, Pfizer, Pflüger, Pharming, Pierre Fabre, Regeneron, Takeda, Regeneron, Sanofi-Genzyme, and UCB Pharma. Elisabeth Wurzer and Hans Joachim Hutt are employees of LEO Pharma GmbH. Ralph von Kiedrowski provides with his service company CMS3 GmbH consulting services, registry research, activities as an investigator in interventional and non-interventional studies, other services and scientific lectures for the following companies: AbbVie, ALK Scherax, Almirall Hermal, Amgen, Beiersdorf Dermo Medical, Biofrontera, Biogen, BMS, Boehringer Ingelheim, Celgene, DermaPharm, Foamix, Gilead, Hexal, Janssen-Cilag, LEO Pharma, Lilly Pharma, Medac, Menlo, MSD, Mylan/Viatris, Novartis, Dr. R. Pfleger, Pfizer, Regeneron, Sanofi, Stallergens, Stiefel GSK, Tigercut, and UCB.

## Funding Sources

This study, including editorial support by Content Ed Net, Germany, in line with Good Publishing Practice (GPP-3) guidelines, was funded by LEO Pharma GmbH.

## Author Contributions

Petra Staubach and Hans Joachim Hutt contributed to conceptualization and design of the non-interventional study; data acquisition was undertaken at the clinical centres by Petra Staubach and Ralph von Kiedrowski, among other clinical centres; and formal analysis of the data was managed by Elisabeth Wurzer together with the CRO Anfomed GmbH. Petra Staubach and Elisabeth Wurzer worked with the medical writer to prepare the first draft of the manuscript in line with Good Publishing Practice (GPP-3) guidelines. Petra Staubach, Elisabeth Wurzer, Hans Joachim Hutt, and Ralph von Kiedrowski contributed comments and helped revise each version of the manuscript and read and approved the final version of the manuscript as submitted.

## Data Availability Statement

The data generated for this study and relevant documents including study documents (e.g., study report, study protocol, statistical analysis plan) will be made available to interested parties after publication of the manuscript in a peer-reviewed journal (providing that regulatory activities are complete) in response to requests directed to the corresponding author.

## Figures and Tables

**Table 1:** Patient characteristics and sociodemographic data

Sociodemographic characteristics (*n* = 217)	
Mean ± SD age, years	49.6±17.2

Gender: female/male, *n* (%)	132/85 (60.8/39.2)

Marital status: single/married/other, ^a^*n* (%)	69/125/23 (31.9/57.9/10.2)

Education: secondary school certificate/high school certificate/university degree/other, ^b^*n* (%)	144/37/30/6 (67.3/17.3/14.0/2.8)

Occupation: working or studying/trainees/pensioners/house-wife or -husband/other, ^c^*n* (%)	136/7/53/14/7 (63.0/3.2/24.5/6.5/3.2)

Psoriasis medical history (*n* = 217)	

Mean ± SD duration of psoriasis [years]	13.1±13.6

First skin site affected by psoriasis [scalp/other] (%)	118/99 (54.9/45.1)

Other skin sites affected by psoriasis (>5% patients) in 217 patients with scalp psoriasis	Arms (53.5%), trunk (48.4%), legs (46.5%), hands (18.9%), and feet (8.8%)

Mean ± SD PASI at baseline [points]	6.8±6.6

Mean ± SD overall BSA at baseline [%]	7.8±7.1

Scalp psoriasis at baseline [Scalp-PGA, % points]	Mild, 13.4%; moderate, 57.1%; severe, 29.5%

Mean ± SD Scalp-BSA at beginning of observation [%]	32.7±19.3

Previous topical therapy [yes/no] (%)	104/113 (47.9/52.1) Most common therapies were corticosteroids (*n* = 70, 32.3%) which included clobetasol (*n* = 29, 13.4%), Cal/BD (*n* = 21,9.7%), and BD alone (*n* = 14, 6.5%)

Comorbidities (*n* = 217)	

All patients with concomitant diseases, *n* (%)	91 (41.9)

Hypertension, *n* (%)	55 (60.4)

Obesity, *n* (%)	21 (23.1)

Diabetes mellitus, *n* (%)	18 (19.8)

BSA, body surface area; PASI, Psoriasis Area and Severity Index; PGA, Physician Global Assessment.

a Widowed/divorced/living alone and one not recorded.

b No educational qualifications applicable or documented.

c Unemployed, unable to work, or not known.

**Table 2:** Mean ± SD of patients reported scalp psoriasis symptom scores during the course of treatment (LOCF): flaking, erythema, thickening, and visibility (rated using 5-point scale of 0 = not at all to 4 = very), degree of spread (6-point scale of 0 = 0% to 5 = 90–100%), and itch (rated using a NRS ranging from 0 = no itch to 10 = maximum itch)

Time of assessment	Scalp flaking (*n* = 192)	Scalp erythema (*n* = 188)	Scalp thickening (*n* = 183)	Lesion visibility *(n* = 181)	Psoriasis spread (*n* = 194)	Itch (*n* = 194)
Baseline	3.05±1.00	2.75±0.93	2.20±1.30	2.35±1.30	2.59±0.97	6.11±2.21
After 3 days' treatment	2.46±1.12^§^	2.19±1.05^§^	1.77±1.26^§^	1.96±1.29^§^	2.44±1.06*	4.90±2.36^§^
After 1 week's treatment	1.76±1.16^§^	1.68±1.05^§^	1.30±1.21^§^	1.48±1.25^§^	2.07±1.06^§^	3.73±2.41^§^
After 2 weeks' treatment	1.32±1.26^§^	1.28±1.05^§^	0.89±1.14^§^	1.07±1.22^§^	1.65±1.15^§^	2.84±2.63^§^
After 4 weeks' treatment	0.97±1.04^§^	0.99±0.95^§^	0.57±0.90^§^	0.66±0.94^§^	1.27±1.09^§^	1.96±2.19^§^

*p* values versus baseline:

§ <0.001;

* = 0.004.
